# Endogenous Endophthalmitis Associated With COVID-19: A Systematic Review on Its Incidence, Risk Factors, Causative Organisms, and Prognosis

**DOI:** 10.7759/cureus.70523

**Published:** 2024-09-30

**Authors:** Abdulaziz M Alshehri

**Affiliations:** 1 Ophthalmology, Taif University, Taif, SAU

**Keywords:** bacterial endogenous endophthalmitis, covid-19, covid-19-related immune dysregulation, fungal endogenous endophthalmitis, intraocular infection

## Abstract

Endogenous endophthalmitis (EE) is a rare but severe intraocular infection resulting from hematogenous dissemination of microorganisms. During the COVID-19 pandemic, there has been a notable increase in EE cases. This literature review aims to evaluate studies focusing on EE associated with COVID-19 to elucidate its pathogenesis and optimize patient management strategies. A thorough search was conducted across PubMed, ScienceDirect, and Google Scholar for relevant research. The surge in EE cases during the COVID-19 pandemic is likely linked to alterations in immune status and systemic comorbidities exacerbated by the virus and its treatments. Notably, *Candida albicans* and *Aspergillus* species emerged as the predominant fungal pathogens in these cases. The findings suggest that the increased incidence of EE is associated with immune dysregulation and increased vulnerability of COVID-19 patients, particularly those with severe diseases or undergoing immunosuppressive treatments. Early diagnosis with timely and effective treatment is crucial for improving patient outcomes. Regular ophthalmic evaluations for hospitalized COVID-19 patients are strongly advised to detect and address ocular complications early.

## Introduction and background

Endogenous endophthalmitis (EE) is a rare but serious intraocular infection resulting from the hematogenous spread of organisms from a distant primary source of infection [1,2]. It accounts for 2-8% of all cases of endophthalmitis and is associated with immunosuppression, intravenous drug use, indwelling catheters, HIV/AIDS, diabetes mellitus, malignancy, and other chronic diseases [3-8]. The most common pathogens include *Staphylococcus aureus*, *Klebsiella pneumoniae*, *Candida albicans,* and *Aspergillus species* [5,9].

Since the emergence of COVID-19, cases of EE have been increasingly reported in patients with severe SARS-CoV-2 infection [10-12]. COVID-19 can cause immune dysregulation and hyperinflammation, increasing susceptibility to secondary infections. Bacterial coinfection, in hospitalized SARS-CoV-2-infected patients, has been reported to be up to 7%, with a higher incidence of approximately 14% in people who need intensive care. Fungal coinfection has also been noted with higher occurrence than in non-COVID patients [13-16]. Reported ophthalmic manifestations of COVID-19 are a handful, not limited to conjunctivitis, keratoconjunctivitis, and episcleritis. There are more serious in nature, such as central retinal vein and artery occlusion, acute retinal necrosis, optic neuritis, neuro-retinitis, ptosis, sixth cranial nerve palsy, dacryoadenitis, orbital cellulitis, and endophthalmitis [17-20]. However, SARS-CoV-2 RNA is rarely detectable in ocular fluids, even in severe diseases [21-23].

According to recent case reports, bacteria like *Klebsiella pneumoniae* and fungi like* Aspergillus* and *Candida* can produce EE, which is linked to severe COVID-19. This infection is probably exacerbated by immunosuppression and comorbidities such as diabetes. Proposed pathways include nosocomial transmission in the intensive care unit (ICU) and COVID-19-induced immunosuppression, which facilitates the spread of latent pathogens [24-28]. To fully understand the pathophysiology of post-COVID-19 EE and develop effective preventative and therapeutic measures, further research is essential. However, a high index of suspicion is warranted in hospitalized COVID-19 patients presenting with eye complaints, particularly those in ICUs. Early diagnosis and aggressive treatment are key to preserving vision in this devastating complication.

The goal of this review is to comprehend the course and mode of action of EE by compiling the results of all case reports of the disease since the COVID-19 pandemic. This aids in comprehending the pathophysiological characteristics and optimal care for individuals following COVID-19.

## Review

Methodology

Search Strategy

A comprehensive literature search was conducted using PubMed, ScienceDirect, and Google Scholar databases to identify relevant studies written in English on EE associated with COVID-19 published between January 2020 and June 2023 were considered for inclusion. This timeframe reflects the period from the onset of the COVID-19 pandemic to the most recent data available at the time of review.

**Figure 1:**
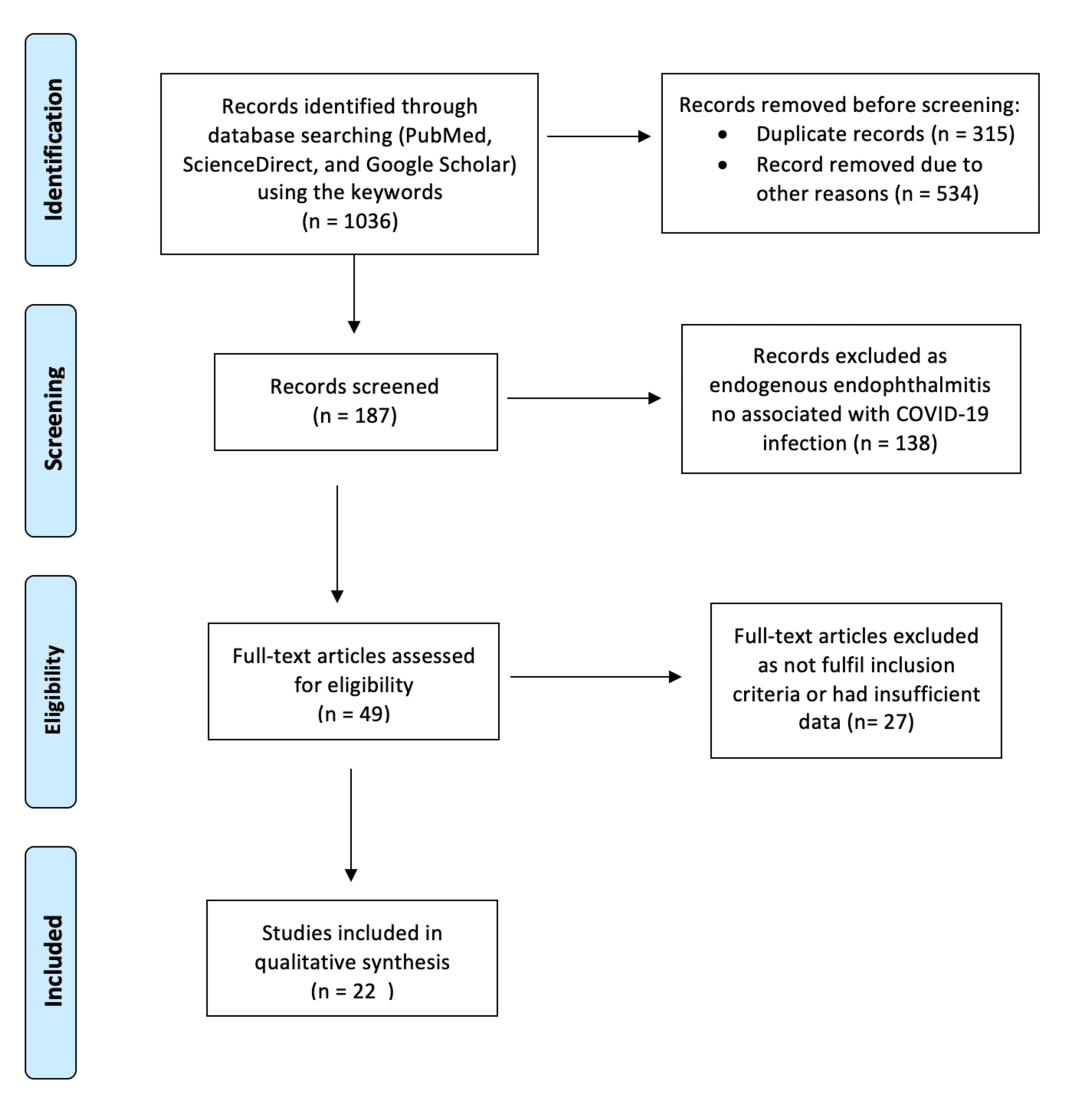
PRISMA flowchart of the studies included articles were excluded based on the eligibility criteria. PRISMA: Preferred Reporting Items for Systematic Reviews and Meta-Analyses

The search terms included “endogenous endophthalmitis," “intraocular infection," “COVID-19," “SARS-CoV-2," and “coronavirus," combined using Boolean operators. All the searches were performed according to Preferred Reporting Items for Systematic Reviews and Meta-Analyses (PRISMA) guidelines (Figure 1).

Selection Criteria

Research reports were considered eligible only if they fulfilled the inclusion criteria. Each article had to meet all of the following inclusion criteria: (1) the patient was affected with COVID-19 either at present or in the recent past less than six months; (2) the patient was specifically diagnosed with EE. We included clinically diagnosed COVID-19 cases (both culture-positive and culture-negative) because this allowed for the inclusion of more studies, which increased the power for analyzing this relatively uncommon complication.

After extensive research across all databases, this search yielded approximately 1,036 results, of which only 187 were relevant. After applying our inclusion and exclusion criteria, we retrieved 22 references, which included 15 case reports, five case series, and a single original research article. The papers were then evaluated based on their level of evidence, and the full text of each article was reviewed.

Results

This comprehensive analysis, which encompasses 22 studies [12,25-44], provides insights into a total of 87 patients and 109 eyes (Table 1). The mean age of patients across the studies was 49.8 ± 6.8 years, with a predominant male representation (65%). Approximately two-thirds of the study participants had diabetes. The majority of the studies (64%) originated from the Indian subcontinent, while the USA and Iran each contributed two published studies. In addition, Egypt, South Africa, Sri Lanka, and Turkey each had one study. Among the 22 published studies, 68% (n = 15) were case reports, primarily from 2021 (60%) and 2022 (40%). These reports described one-fifth of the patients affected in both eyes. The majority of patients were observed in a single-specialty eye hospital or clinic, accounting for about two-thirds of the cases. The remaining one-third of patients were treated in multi-specialty hospitals that managed a range of concurrent ailments (Table 1).

**Table 1:** Studies described endogenous endophthalmitis cases in patients with recent exposure to COVID-19

Reference	Type of study	Setting	Number of subjects	Number of eyes	Mean age	Male ratio	Diabetes percentage	Year	Place	Organism	COVID-19 infection
Nayak et al. [12]	Research article	Single-specialty hospital	24	33	53.6	70.83%	87%	2021	India	Fungal 78%, bacterial 14%	2-8 weeks prior
Jain et al. [24]	Case report	Single-specialty hospital	1	1	66	100%	100%	2022	India	Fungal	1 week prior
Fayed et al. [25]	Case report	Single-specialty hospital	2	2	59.5	50%	100%	2022	Egypt	Fungal	1-2 months prior
Crane et al. [26]	Case report	Multi-specialty hospital	1	1	35	100%	NA	2021	USA	Bacterial	Concurrent
Bilgic et al. [27]	Case report	Single-specialty hospital	3	3	NA	NA	NA	2021	India	Bacterial	Concurrent or recent
Sanjay et al. [28]	Case report	Single-specialty hospital	1	2	47	100%	0	2022	India	Bacterial	3 weeks prior
Murthy et al. [29]	Case report	Single-specialty hospital	1	1	56	100%	0	2022	India	Fungal	1 month prior
Nakhwa [30]	Case report	Multi-specialty hospital	1	1	45	100%	NA	2021	India	Fungal	1 month prior
Sahu et al. [31]	Case report	Single-specialty hospital	5	5	NA	NA	NA	2021	India	Fungal	Within 1 month
Mehta et al. [32]	Case report	Multi-specialty hospital	2	4	54.5	100%	100%	2022	India	Fungal	Within 1 week
Khatwani et al [33]	Case report	Single-specialty hospital	7	9	61.3	71%	57%	2021	India	Fungal 85.7%, bacterial 12.5%	Within 1 month
Agarwal et al. [34]	Case report	Multicentric	6	8	NA	NA	67%	2022	India	Fungal 33%, bacterial 33%	Within 1 month
Shroff et al. [35]	Case report	Single-specialty hospital	5	7	50	100%	40%	2021	India	Fungal 71.4%	3 weeks prior
Goyal et al. [36]	Case report	Multi-specialty tertiary care hospital	7	1	42	100%	NA	2021	India	Fungal	4- 6 weeks prior
Kamath et al. [37]	Case report	Multi-specialty hospital	1	1	49	100%	100%	2021	India	Bacterial	1 week prior
Brotherton et al. [38]	Case report	Multi-specialty hospital	1	1	34	100%	NA	2021	USA	Bacterial	1 week prior
Deepa et al. [39]	Case report	Single-specialty hospital	1	1	50	100%	100%	2022	India	Fungal	1 month prior
Namvar et al. [40]	Research article	Single-specialty hospital	14	24	49.57	35.7%	57.15%	2022	Iran	Fungal 42%	Within 1 month
Letsoalo and Mathebula [41]	Case report	Private hospital	1	1	55	100%	100%	2022	South Africa	Bacterial	1 week prior
Kaluarachchi and Abeykoon [42]	Case report	Single-specialty hospital	1	1	62	0	100%	2022	Sri Lanka	Fungal, cytomegalovirus	1 week prior
Kaderli et al. [43]	Case report	Single-specialty hospital	1	1	61	0	0	2022	Turkey	Fungal	1 month prior
Zibaeenezhad et al. [44]	Case report	Multi-specialty hospital	1	1	66	100%	100%	2022	Iran	Fungal, cytomegalovirus	1 week prior

Patients in the studies either had a concurrent COVID-19 infection or had experienced COVID-19 within the past two months. About 50% of these patients had been infected with COVID-19 one month prior. In addition, 18% of the patients either had concurrent infections or had been infected within one week (as shown in Table 1).

Among the 109 eyes examined in 87 patients, the predominant cause of EE was identified as fungal (77%) (Table 2). Eleven out of the 22 studies (comprising 40 patients and 48 eyes) reported the exclusive presence of fungal elements. While not all cases had a confirmed fungal organism isolated from the vitreous sample, the positive response to empirical antifungal treatment led to the diagnosis of fungal endophthalmitis. Even though the majority of eyes were impacted by fungal infections, bacterial infections were reported at a rate of (27%). Bacteria were identified in six studies (eight patients) [26-28,37,38,41]. The commonly isolated bacterial species were *Pseudomonas aeruginosa*, *Klebsiella pneumoniae*, *Staphylococcus aureus,* and methicillin-resistant *Staphylococcus aureus* (MRSA). Other bacteria included *Escherichia* spp. and gram-positive *Streptococcus*.

**Table 2:** Types of microorganisms and associated risk factors for endogenous endophthalmitis in COVID-19-affected patients ICU: intensive care unit, MRSA: methicillin-resistant *Staphylococcus aureus*

References (publication date and place)	Causative agent	Number of studies	Number of subjects	Risk factors
Brotherton et al., 2021 [38] (USA) and Bilgic et al., 2021 [27] (India)	Bacterial – MRSA	2	2	Intravenous drug user bacteremia use of corticosteroids in the management of COVID-19 infection
Crane et al., 2021 [26] (USA) and Bilgic et al., 2021 [27] (India)	Bacterial – *Klebsiella pneumoniae*	2	2	use of corticosteroids in the management of COVID-19 infection in diabetic and immunocompromised patients in the setting of COVID-19 infection
Sanjay et al., 2022 [28] (India) and Letsoalo and Mathebula, 2022 [41] (South Africa)	Bacterial - *Pseudomonas aeruginosa *	2	2	COVID-19 infection in patients with comorbidities, intensive care unit (ICU) admission, and prolonged use of systemic corticosteroids.
Agarwal et al., 2022 [34] (India)	Bacterial - *Staphylococcus aureus*	1	2	67% diabetic patients. COVID-19 patients with a history of hospitalization and prolonged use of systemic corticosteroids and multiple comorbidities
Mehta et al., 2022 [32] (India), Agarwal et al., 2022 [34] (India), Shroff et al., 2021 [35] (India), Namvar et al., 2022 [40] (Iran), and Kaderli et al., 2022 [43] (Turkey)	Fungal – *Candida albicans* species	5	22	Elderly, COVID-19 patients with comorbidities and ICU admission who received systemic corticosteroids.
Jain et al., 2022 [24] (India), Fayed et al., 2022 [25] (Egypt), Sahu et al., 2021 [31] (India), and Khatwani et al., 2021 [33] (India)	Fungal – Aspergillus species	4	15	ICU admission, comorbidities and use of corticosteroids in management of COVID-19 infection. The immunocompromised state in COVID-19 predisposes to endophthalmitis.
Deepa et al., 2022 [39] (India)	Fungal – cryptococcus	1	1	Prolonged hospitalization, ICU admission, and systemic corticosteroid in the setting of COVID-19 infection
Murthy et al. 2022 [29] (India)	Fungal - Fusarium	1	1	Systemic corticosteroid in the setting of COVID-19 infection
Kaluarachchi and Abeykoon 2022 [42] (Sri Lanka), and Zibaeenezhad et al., 2022 [44] (Iran)	Mixed organisms	2	2	ICU admission and systemic corticosteroid in the setting of COVID-19 infection

In five studies, a mixture of organisms was noted [12,33,34,42,44]. Nayak et al. [12] assessed 33 eyes in 24 patients, revealing that 78% of the cases were attributed to fungal endophthalmitis, with viral or bacterial origins accounting for less than 20%. Among the bacterial cases, 4% were identified as having gram-negative bacteria among the vitreous isolates.

The most common viral pathogen was cytomegalovirus, which was confirmed by the vitreous sample PCR [42,44] and seen as superinfection or coinfection in those with fungal endophthalmitis, especially *Candida* species. The other predominant fungal infection was caused by *Aspergillus* spp., *Fusarium*, and *Cryptococcus* were also found more commonly in immunocompromised patients or those with extensive systemic corticosteroid therapy as part of COVID management (Table 2).

In our review, we found that patients either presented with new-onset ocular symptoms or initially sought treatment from tertiary ophthalmology centers before receiving specialized eye care. Early diagnosis and prompt treatment are crucial, as they significantly improve prognosis. Our analysis revealed that 74% of the eyes underwent diagnostic or therapeutic pars plana vitrectomy (PPV) along with intra-vitreal and systemic antimicrobials. By contrast, 23% of the patients received only intra-vitreal and systemic antimicrobials, while 3% received only systemic medications. Despite these interventions, final visual acuity was generally poor. Only one-third of the patients achieved visual acuity better than 20/200. Furthermore, 9.3% of the patients either required evisceration or developed phthisis bulbi after the infection resolved.

Discussion

EE arises from the hematogenous spread originating from a septic focus, such as an embolus. If left untreated or inadequately treated, a localized infection can extend from the vitreous cavity to the ocular coats, potentially leading to pan-ophthalmitis or orbital cellulitis [45-47]. Screening for EE is crucial, especially in high-risk COVID-19 patients who present with eye symptoms, such as decreased vision, floaters, and redness, even up to three months after COVID-19 recovery, to ensure early diagnosis and treatment [12]. Boontantrapiwat et al. reported a case of a 64-year-old woman developing endophthalmitis following a COVID-19 infection, progressing to pan-ophthalmitis despite receiving topical and intravenous antibiotics [48]. The imminent threat to vision and the risk of progressive permanent complications underscore the importance of early diagnosis and aggressive therapy. Endophthalmitis should be diagnosed based on the clinical picture although it may have a subacute course, especially in sick COVID-19 patients, causing delayed presentation and poorer prognosis. Aqueous and/or vitreous cultures, with or without additional PCR for pan-bacterial and pan-fungal primers, can assist in isolating microorganisms and contribute to specifying the appropriate medication. In our review, the diagnosis of COVID-19 infection relied primarily on patient history and previous reports, with only three patients testing positive for RT-PCR. Crane documented patients testing positive for COVID-19 during the onset of ocular symptoms [26]. Common presenting symptoms included redness, floaters, blurred vision, or pain, typically in one eye and rarely in both. Blurred or loss of vision was the most frequently reported symptom. Clinicians need to maintain a high suspicion for endophthalmitis, particularly in patients with a history of COVID-19 infection and have eye symptoms, considering other corneal or retinal involvements. Fundus examination should form a part of the management protocol for patients being treated for post-COVID-19 complications [33].

When EE is suspected, particularly in the presence of predisposing inflammatory conditions like COVID-19, it is advisable to obtain blood, urine, and vitreous cultures before initiating empirical therapy. Furthermore, early initiation of appropriate treatment is crucial to contain the spread of infection, involving the use of antimicrobial medications intravenously and/or intravitreally, along with topical eye medications [49]. The predominant bacterial pathogens are typically associated with the *Staphylococcus *and *Streptococcus* groups [26]. However, our study revealed a higher prevalence of *Pseudomonas aeruginosa* and *Klebsiella pneumoniae*, with the remaining cases involving gram-negative bacteria [8]. It is noteworthy that even in non-COVID patients, *Candida *is a common cause of EE. Namvar established that the incidence and symptoms of endophthalmitis in patients with or without COVID-19 do not significantly differ, and their progression poses an equal level of affection [40]. Nevertheless, patients with a history of COVID-19 are more susceptible due to their immunocompromised status and the use of extensive immunosuppressants.

Therapeutic intervention is often urgent and may involve intravitreal medications or PPV. Early medical and surgical intervention for EE in the setting of COVID-19 infection can have good outcomes [26]. Comorbidities, especially diabetes, pose a significant risk for developing septic foci and are associated with a poor response to treatment. Prolonged hospitalization, ICU admission, and indwelling urinary catheters are additional factors that exacerbate the situation. Broad-spectrum antibiotics play a crucial role in eliminating bacteria and preventing sepsis, confining the focus of infection, and averting systemic complications. However, their extensive use can disrupt the balance of commensal bacteria, allowing the proliferation of yeast and other fungi [33]. Prolonged antibiotic use, especially when coupled with immune suppressants such as corticosteroids, can contribute to fungal infections in COVID-19 patients. Viral coinfections may also arise due to similar reasons.

In the reviewed studies, some patients demonstrated improved visual outcomes following intraocular administration of antibiotics or antifungals and vitrectomy, complemented by systemic therapy. However, cases where the diagnosis was initially missed despite therapy exhibited poor anatomical and functional outcomes.

The findings of this study are subject to several limitations. As previously mentioned, EE is an uncommon condition, which complicates the ability to conduct randomized controlled trials. This limitation forces reliance on studies with small sample sizes, often lacking control groups and primarily consisting of case reports and case series. In addition, the heterogeneity observed in the included studies, characterized by variations in diagnostic criteria, treatment protocols, and healthcare settings across different regions, can lead to discrepancies in outcomes. Notably, a significant portion of the studies originates from the Indian subcontinent, reflecting unique healthcare practices, cultural influences, population characteristics, and access to treatment that may not be representative of other regions.

Further research is needed to investigate EE cases secondary to causes other than COVID-19 in various geographic locations. Such studies would provide a broader understanding of the condition and allow for meaningful comparisons with findings related to COVID-19.

## Conclusions

During the COVID-19 pandemic, there has been a notable increase in EE cases, particularly among hospitalized elderly diabetic patients who received systemic steroids. This condition, associated with high rates of mortality and poor outcomes, emphasizes the importance of vigilant screening and early detection of ocular manifestations, especially in high-risk individuals. The pandemic has led to a rise in fungal infections, predominantly caused by *Candida* spp. and *Aspergillus* spp., as well as a shift toward gram-negative bacterial infections. It is essential for treating physicians to be aware of these evolving trends to optimize patient management and outcomes in the context of COVID-19.
